# Supplementary material for: Point mutations in Arf1 reveal cooperative effects of the N-terminal extension and myristate for GTPase-activating protein catalytic activity
Source: PLoS One. 2024 Apr 4;19(4):e0295103. doi: 10.1371/journal.pone.0295103 (PMC10994351; doi:10.1371/journal.pone.0295103)
Supplement: S1 Text — (DOCX) [file pone.0295103.s001.docx]

**Supporting Information:**

Point mutations in Arf1 reveal cooperative effects of the N-terminal region and myristate for GTPase-activating protein catalytic activity

Eric M. Rosenberg Jr.^1,^^¶^, Xiaoying Jian^1,¶^, Olivier Soubias^2^, Rebekah A. Jackson^2^, Erin Gladu^1^, Emily Andersen^1^, Lothar Esser^3^, Alexander J. Sodt^4^, Di Xia^3^, R. Andrew Byrd^2^, Paul A. Randazzo^1*^

**Affiliations:**

^1^Laboratory of Cellular and Molecular Biology, Center for Cancer Research, National Cancer Institute; Bethesda, MD, USA.

^2^Section of Macromolecular NMR, Center for Structural Biology Laboratory, Center for Cancer Research, National Cancer Institute; Frederick, MD, USA.

^3^Laboratory of Cell Biology, Center for Cancer Research, National Cancer Institute; Bethesda, Maryland, USA.

^4^Unit of Membrane Chemical Physics, *Eunice Kennedy Shriver* National Institute of Child Health and Human Development; Bethesda, MD, USA

*Corresponding author

E-mail: randazzp@mail.nih.gov

^¶^These authors contributed equally to this work

S1 Text. Discussion regarding the decreased apparent maximum GTP hydrolysis observed with [L8A/F13A]myrArf1.

The apparent change in maximum could be due to a change in background signal, with a number of possible explanations. A time course of the GAP reaction using WT myrArf1 and the double mutant catalyzed with saturating amounts of ASAP1_PZA_ suggested that the reaction occurs with similar kinetics, yet the double mutant still plateaued at a lower level than WT (S3A Fig), perhaps indicative of a fraction of GTP-bound Arf that was not accessible to the GAP. For WT myrArf1 and most mutants, the ratio of bound GDP to bound GTP did not change over a 40 minute incubation at 30ºC in the absence of GAP, while the ratio did change for [L8A/F13A]myrArf1 (S3B Fig). Although intrinsic GTPase activity in this mutant is possible, an equally plausible explanation is that this mutant could exchange nucleotide in the presence of 1 mM Mg^2+^ (which is typically used to quench exchange after loading [α^32^P]GTP) and there was a small amount of contaminating nucleotidase, a common occurrence in proteins expressed in and purified from bacteria [37]. We also note that both [F13A] and [L8A/F13A]myrArf1 had a greater fraction of GDP-bound Arf at all time points, whereas that was not observed with WT or other myrArf1 mutants (S3B Fig). Altogether, characterization of these mutants will require further investigation.
