# Supplementary figures and images for: Point mutations in Arf1 reveal cooperative effects of the N-terminal extension and myristate for GTPase-activating protein catalytic activity
Source: PLoS One. 2024 Apr 4;19(4):e0295103. doi: 10.1371/journal.pone.0295103 (PMC10994351; doi:10.1371/journal.pone.0295103)

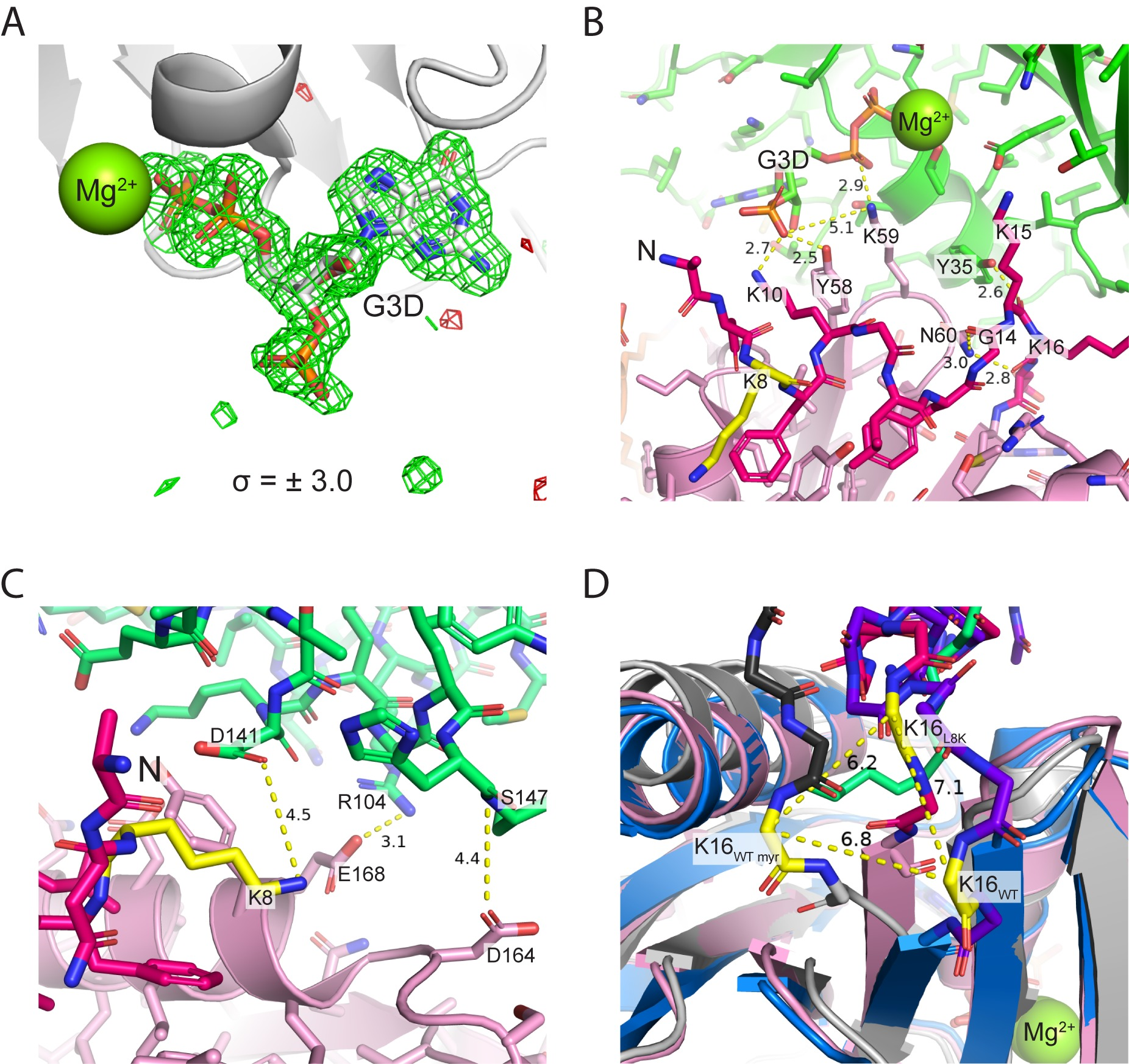

Supplement: S1 Fig — (A) Polder OMIT map [51] of the atoms composing guanosine-3’-monophosphate-5’-diphosphate (G3D) within the [L8K]Arf1•G3D crystal structure. Green and red mesh corresponds to positive and negative mFobs − DFmodel OMIT difference density contoured at 3σ. (B) Interswitch region crystal contacts between monomers within the [L8K]Arf1•G3D crystal structure. The interswitch region of one monomer (pink) is adjacent to an alpha helix between the P-loop and switch I in the G domain of another monomer (green). Crystal contact regions are emphasized with yellow dashed lines, and distances in Angstroms are shown. N-terminus and contact residues are labeled, and the L8K mutation is shown in yellow. (C) G5 motif crystal contacts between monomers within the [L8K]Arf1•G3D crystal structure. The G5 motif of one monomer (pink) is adjacent to an alpha helix towards the C-terminal end of the G domain of another monomer (green). Residues and crystal contacts are emphasized as in (B). Note that the L8K mutation residue (yellow) is near D141 in this same symmetry mate. (D) Hinge regions between N-terminal extension and G domains in Arf1 structures. The coloring of each structure is consistent with the colors used in Fig 1. The backbone atoms of K16 are shown in yellow and labeled, and the distances between K16 α-carbons are shown in yellow dashed lines. Distances are in Angstroms. (TIF) [file pone.0295103.s002.tif]

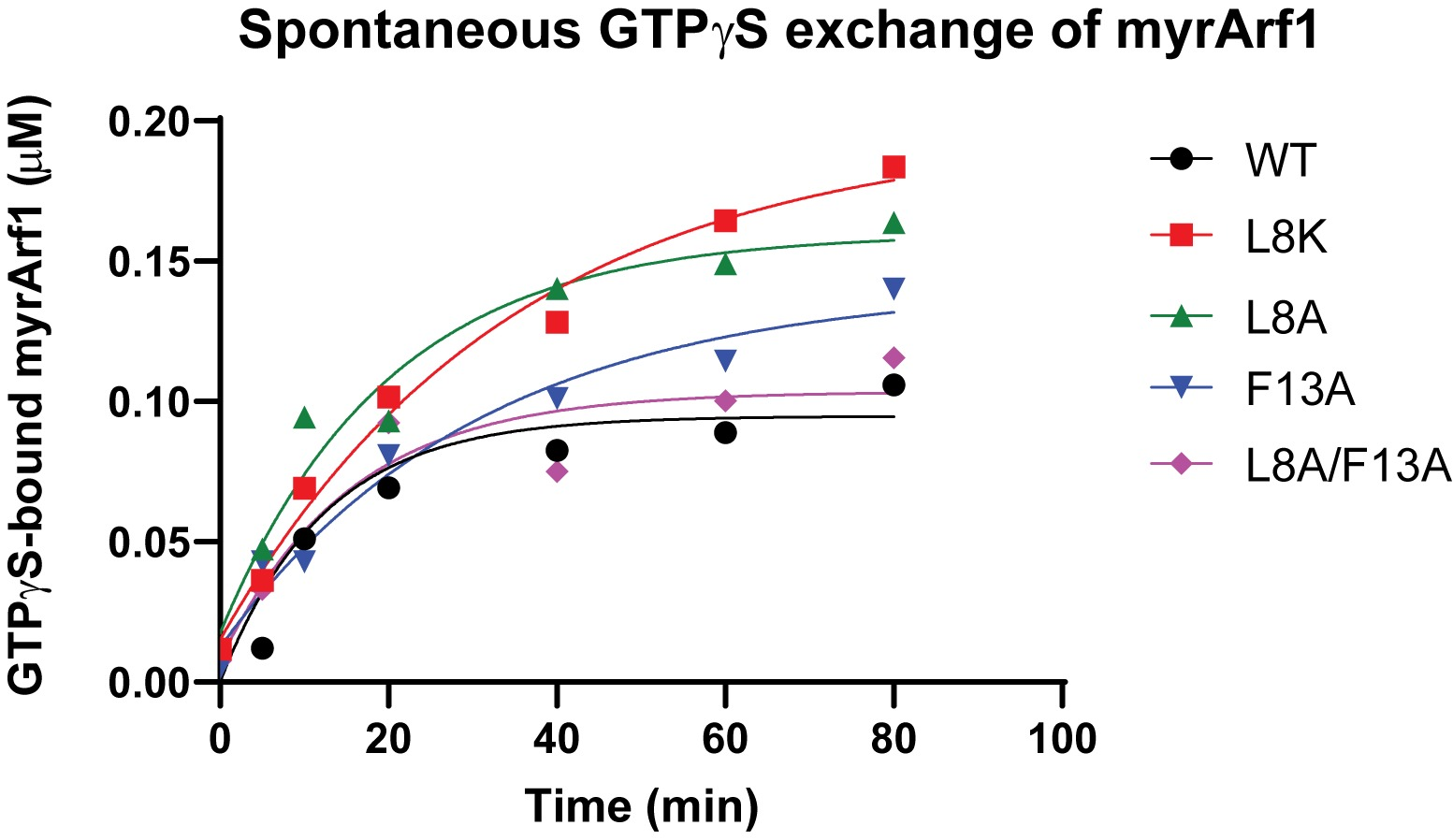

Supplement: S2 Fig — For these assays, 0.5 μM myrArf1 constructs was added to a reaction with low (1–10 μM) Mg2+ to promote exchange, as well as [35S]GTPγS and LUVs. After the indicated period of time, the fraction of myrArf1 bound to [35S]GTPγS was measured. Data shown are a representative example from multiple experiments. (TIF) [file pone.0295103.s003.tif]

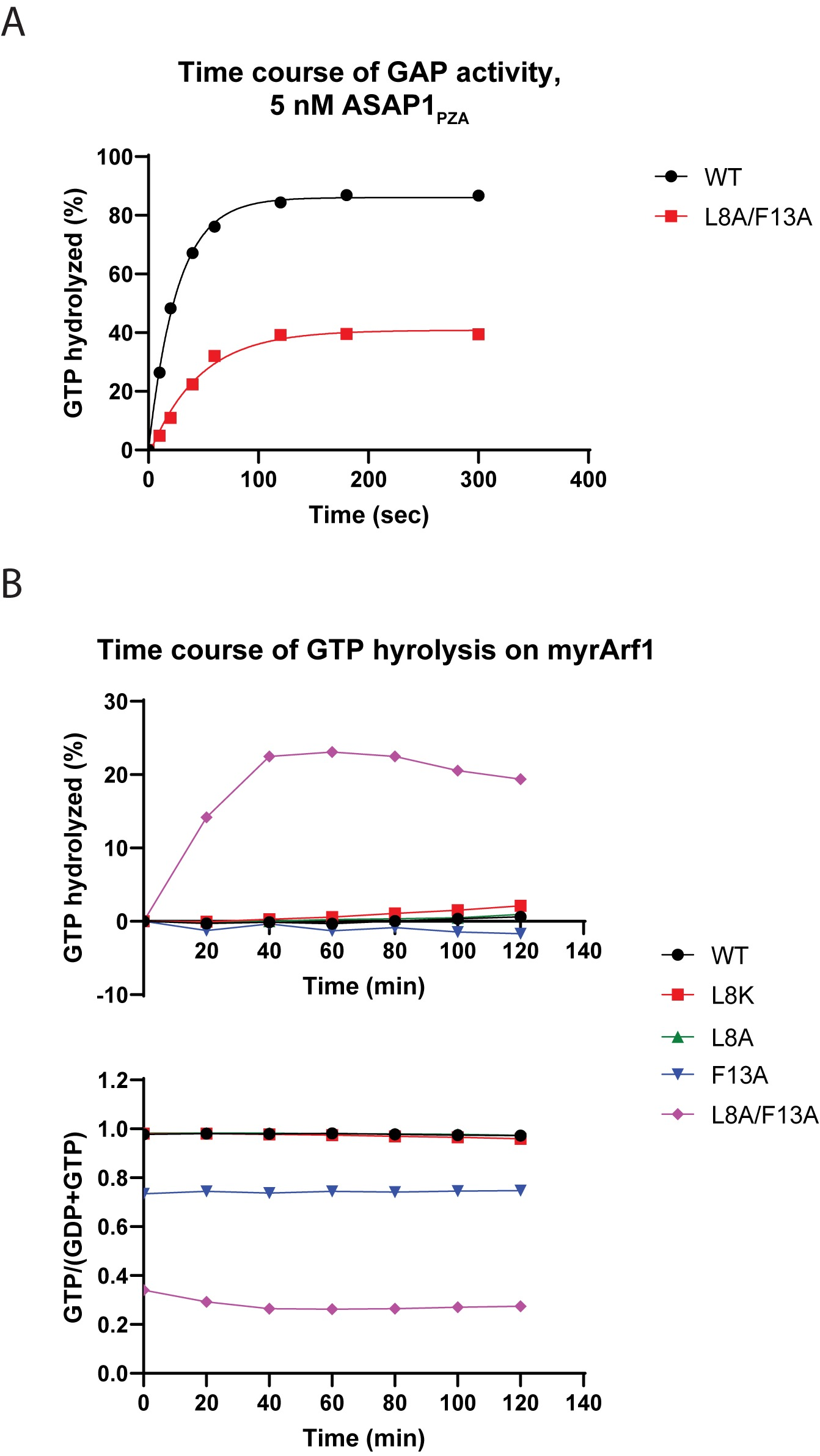

Supplement: S3 Fig — (A) Time course of Arf GAP activity with high concentrations (5 nM) of ASAP1PZA using WT or [L8A/F13A]myrArf1•GTP as substrates. For these assays, myrArf1 was loaded with [α32P]GTP, ASAP1PZA was added, the reaction was quenched after the indicated incubation time, and the ratio of [α32P]GDP and [α32P]GTP bound to myrArf1 was measured. Data shown are a representative example from multiple experiments. (B) Time course of spontaneous GTP hydrolysis on myrArf1 constructs. For these assays, myrArf1 was loaded with [α32P]GTP for 30 minutes. Following the indicated periods of time after addition of GAP reaction buffer containing Mg2+ and GTP, the reaction was quenched and the ratio of [α32P]GDP and [α32P]GTP bound to myrArf1 was measured. The panel on top shows the change in GTP hydrolyzed compared to that at time 0 minutes (immediately after 30 minutes of GTP loading then quenching); the panel on bottom is the same data, showing the full ratio of measured GTP over the sum of measured GDP and GTP. Data shown are a representative example from multiple experiments. (TIF) [file pone.0295103.s004.tif]

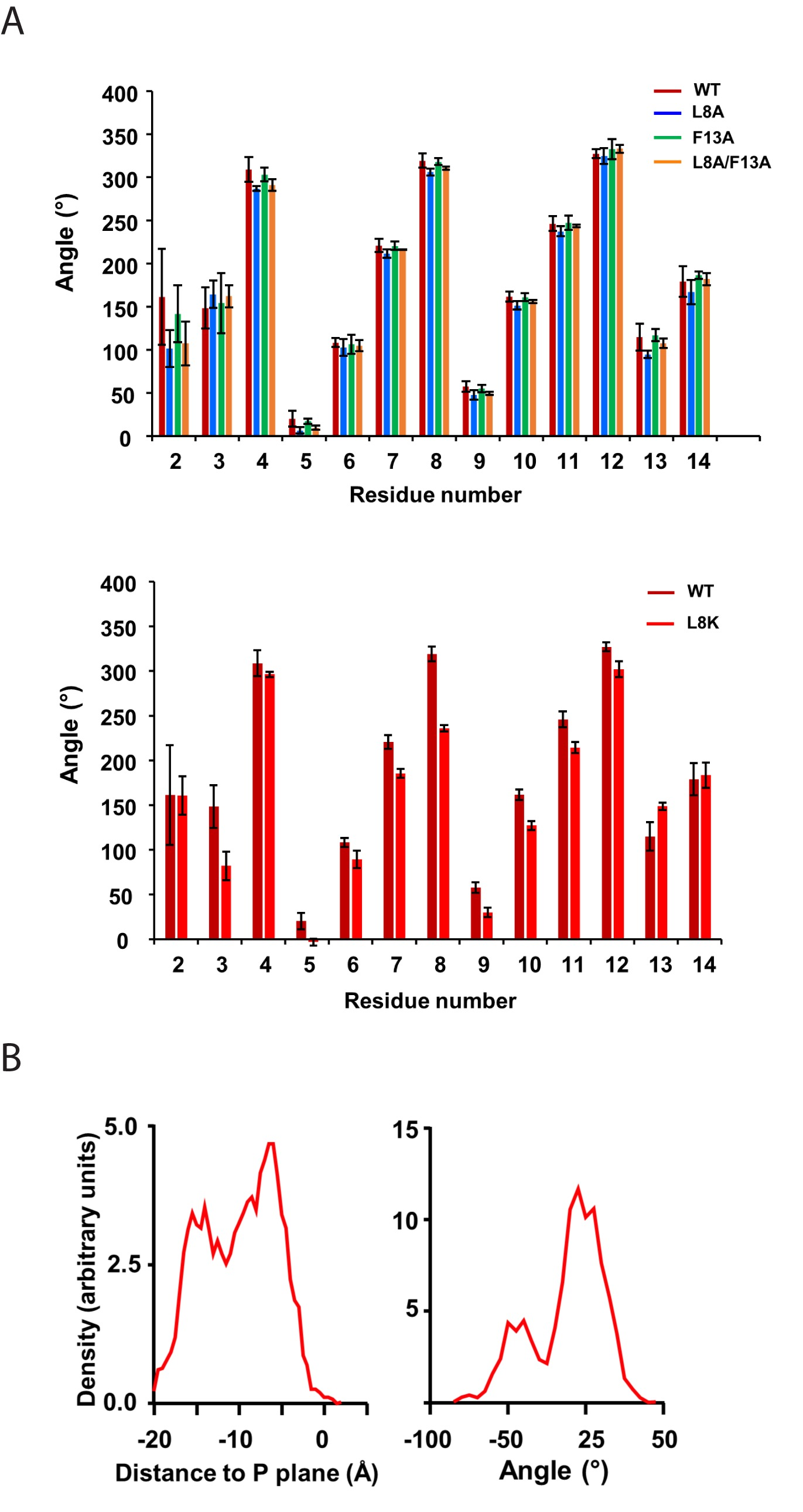

Supplement: S4 Fig — (A) Per residue roll angle for WT myrArf1 peptide and its mutants. WT myrArf1 per residue roll angles are compared to [L8A]myrArf1, [F13A]myrArf1, and [L8A/F13A]myrArf1 (top) or [L8K]myrArf1 (bottom). Roll angles are calculated as the angle between the Calpha-Halpha bond of a residue and the bilayer normal. An angle of 0° corresponds to the Calpha-Halpha bond aligned with the bilayer normal and pointing toward the hydrophobic core of the membrane. (B) Insertion depth (left) and average orientation (right) relative to the monolayer phosphate membrane plane of [L8K]myrArf1 N-terminal peptide. A tilt angle of zero means that the helical axis is parallel to the membrane surface. A negative tilt angle means the peptide is tilted such that the N-terminus is lower than the C-terminus on the z-axis (membrane normal). (TIF) [file pone.0295103.s005.tif]
